# High Dosage Folic Acid Supplementation, Oral Cleft Recurrence and Fetal Growth

**DOI:** 10.3390/ijerph10020590

**Published:** 2013-02-04

**Authors:** George L. Wehby, Têmis Maria Félix, Norman Goco, Antonio Richieri-Costa, Hrishikesh Chakraborty, Josiane Souza, Rui Pereira, Carla Padovani, Danilo Moretti-Ferreira, Jeffrey C. Murray

**Affiliations:** 1 Department of Health Management and Policy, University of Iowa, Iowa City, IA 52242, USA; E-Mail: george-wehby@uiowa.edu; 2 Hospital de Clínicas de Porto Alegre, Porto Alegre, Rio Grande do Sul 90035-903, Brazil; E-Mail: tfelix@hcpa.ufrgs.br; 3 RTI International, Research Triangle Park, NC 27709, USA; E-Mail: ngoco@rti.org; 4 Hospital de Reabilitação de Anomalias Craniofaciais, Bauru, Sao Paulo 17.043-900, Brazil; E-Mail: richieri@usp.br; 5 Department of Epidemiology and Biostatistics, University of South Carolina, Columbia, SC 29208, USA; E-Mail: rishic@mailbox.sc.edu; 6 Centro de Atendimento Integral ao Fissurado Lábio Palatal, Curitiba, Paraná 81.050-000, Brazil; E-Mail: jositwin@yahoo.com.br; 7 Instituto Materno Infantil Prof. Fernando Figueira-CADEFI/IMIP, Recife, Pernambuco 50070-550, Brazil; E-Mail: ruimrpereira@terra.com.br; 8 Hospital Santo Antônio: Obras Sociais Irmã Dulce, Salvador, Bahia 40.415-000, Brazil; E-Mail: carla.mca@uol.com.br; 9 Genetic Counseling Service, São Paulo State University, Botucatu, Sao Paulo 18618-000, Brazil; E-Mail: danilo@ibb.unesp.br; 10 Department of Pediatrics, University of Iowa, Iowa City, IA 52242, USA

**Keywords:** oral clefts, cleft lip, cleft palate, birth defects, folic acid, vitamins, prevention, pregnancy, nutrition, Brazil, NCT00397917

## Abstract

*Objectives*: To evaluate the effects of folic acid supplementation on isolated oral cleft recurrence and fetal growth. *Patients and Methods*: The study included 2,508 women who were at-risk for oral cleft recurrence and randomized into two folic acid supplementation groups: 0.4 and 4 mg per day before pregnancy and throughout the first trimester. The infant outcome data were based on 234 live births. In addition to oral cleft recurrence, several secondary outcomes were compared between the two folic acid groups. Cleft recurrence rates were also compared to historic recurrence rates. *Results*: The oral cleft recurrence rates were 2.9% and 2.5% in the 0.4 and 4 mg groups, respectively. The recurrence rates in the two folic acid groups both separately and combined were significantly different from the 6.3% historic recurrence rate post the folic acid fortification program for this population (*p* = 0.0009 when combining the two folic acid groups). The rate of cleft lip with palate recurrence was 2.9% in the 0.4 mg group and 0.8% in the 4 mg group. There were no elevated fetal growth complications in the 4 mg group compared to the 0.4 mg group. *Conclusions*: The study is the first double-blinded randomized clinical trial (RCT) to study the effect of high dosage folic acid supplementation on isolated oral cleft recurrence. The recurrence rates were similar between the two folic acid groups. However, the results are suggestive of a decrease in oral cleft recurrence compared to the historic recurrence rate. A RCT is still needed to identify the effect of folic acid on oral cleft recurrence given these suggestive results and the supportive results from previous interventional and observational studies, and the study offers suggestions for such future studies. The results also suggest that high dosage folic acid does not compromise fetal growth.

## 1. Introduction

Oral clefts are among the most common birth defects worldwide, with incidence ranging from 1/200 births in Amerindian populations to about ½,500 births in African populations and an intermediate incidence for European populations at about 1/700 births [1]. In addition to the short-term health consequences and healthcare costs due to reduced fetal growth [2] and the need for surgical repair, oral clefts have long-term adverse effects on health requiring dental, speech, and psychosocial interventions and increase hospitalization risks up to adulthood [3]. Oral clefts significantly reduce the quality of life of affected children and their families [4,5].

The etiology of oral clefts is complex involving genetic and environmental factors with both independent and interactive effects. Although much of the genetic etiology, particularly functional and developmental pathways, remain unknown, several genes/loci have been implicated in oral clefts [6,7,8,9], with most compelling evidence for IRF6 and loci near 8q24, MAFB, ABCA4 and VSX1 [8,10]. Several environmental/behavioral factors may play a role particularly smoking, which may have both independent [9] and interactive effects with genetic risk factors [11], and folate/multivitamin use [12]. 

There has been a wide interest in evaluating the role of maternal nutrition particularly the consumption of folate rich diets and use of folic acid supplements in oral cleft recurrence and occurrence. A detailed review of this literature is presented elsewhere [12]. Several observational studies provide supportive evidence for a protective effect of folate and folic acid supplementation on oral cleft occurrence. However, there is still a large uncertainty about the role of folic acid supplementation on occurrence because these observational studies might not entirely account for bias resulting from maternal self-selection into vitamin use and nutritional behaviors, which may be correlated with unobserved health or behavioral characteristics that affect oral cleft risks. 

The risk of oral cleft recurrence, defined as the occurrence of oral clefts in already affected families (*i.e.*, mother is affected or has had a previous child with oral clefts) is higher by about 40 times than overall prevalence in the general population (4–5% *versus* 1/1,000, respectively). Several studies have evaluated the effects of folic acid supplementation, particularly high *versus* low doses on oral cleft recurrence. Most of these studies found significant protective effects of high doses—on average a decrease of 50% in recurrence (see detailed review in [12]). However, unlike the evidence for a preventive effect on neural tube defect (NTD) recurrence [13], the evidence for a preventive effect on oral cleft recurrence is still controversial, mainly because none of the previous interventional studies used a randomized design and as a result were still prone to bias by lacking an appropriate control group. Another limitation of some studies was the challenge involved in evaluating folic acid effects independent of other vitamins.

The Oral Cleft Prevention Program (OCPP) was as a double blinded RCT to assess the effect of high-dose *versus* low-dose folic acid supplementation on oral cleft recurrence among children of Brazilian women [14]. We describe the OCPP’s design and report the findings as of December 2009 after the trial was halted due to lower than expected enrollment and pregnancy rates among study participants. We also compare the recurrence rates in the OCPP to a historic recurrence rate. In addition, we report data on fetal growth outcomes, compliance, and adverse events. The comparisons we present on fetal development outcomes between high and low folic acid supplementation groups are the first from a randomized study in humans. Finally, we provide insights based on the lessons learned from the OCPP for a future study that can provide a definitive answer about the effects of high dose folic acid on oral cleft recurrence.

## 2. Methods

### 2.1. Design

This study aimed at testing the effect of 4 mg folic acid taken daily before pregnancy throughout the first trimester by women who were themselves born with a cleft or had had a prior child with a cleft on recurrence of clefting in a subsequent child compared to taking the currently recommended 0.4 mg dose [14]. A placebo control group was not used, as a low dose of folic acid had already been recommended as a standard vitamin therapy for women during preconception and pregnancy period for prevention of NTDs.

The study had a double-blinded randomized design, where both investigators and participants were blinded to the study group assignment [14]. Participants were randomly assigned before pregnancy to the two study groups of taking 4 mg or 0.4 mg pills of folic acid that were identical in appearance. Randomization occurred at the participant level and was stratified by the study site to ensure a balanced representation in both treatment groups. The study Data Center at RTI International oversaw the randomization and generated the randomization sequence using permuted blocks of random size. The randomization sequence linked the treatment assignment (0.4 mg or 4 mg) to a sequential list of serial numbers that were affixed to the study pill boxes under the supervision of the Data Center. The pillboxes were also numbered in order of dispensing (1, 2, 3, *etc.*). The folic acid pills were specifically manufactured for the study by ATIVUS Pharmaceutical Industries in Brazil. The manufacturer conducted quality control tests for each batch of pills including identification test of the raw folic acid material used in the pills (prior to production), assay tests for the folic acid dosage in the pills, content uniformity tests (to ensure that the dosage is consistent across pills), and dissolution tests to ensure that the pills dissolve adequately in a standard time.

Participants who had a delay in menstruation of 14 days or more at baseline underwent a pregnancy test before randomization; only those with a negative test were randomized [14]. After randomization, all participants were asked to take a single pill of 4 mg or 0.4 mg of folic acid daily throughout the first trimester of pregnancy if they became pregnant or until end of participation in the study if they did not become pregnant. Participants were followed up every two months to check on their health status, provide new pill boxes for the next two months, and collect old pillboxes with any unused pills to measure compliance. During the first two years of the study (between 2004 and 2006), bimonthly follow-ups were completed in person. In later years, bimonthly follow-ups were mostly completed over the phone and study pills delivered to participants through express mail. When participants were available, periodic in-person follow-ups were conducted every six months. Compliance was evaluated by counts of unused pills from returned pillboxes. An average compliance rate per participant was generated from all the returned pillboxes for that participant. Serum and RBC folate levels, evaluated periodically after randomization in 2004–2006 and after 12 months of randomization (or at pregnancy, whichever occurred first) beginning in 2007 also provided another measure of compliance. Folate levels were not communicated with the subjects and were seen and entered by staff not directly involved with the study subjects.

Participants confirmed to be pregnant were advised by the study staff to stop taking the study pills at the end of the first trimester of pregnancy [14]. There were no restrictions on participants taking other supplements and vitamins before or during pregnancy as recommended by their physicians. The study staff attempted to follow pregnant participants periodically (usually every two months) over the phone to check on pregnancy progress. Furthermore, the study staff attempted to maintain contact with the participants’ prenatal care providers to check on pregnancy progress.

After delivery, all live births were followed in-person by the study staff to measure oral cleft status. Mothers also completed an interview with the study staff after delivery either in-person or over the phone to obtain data on perinatal and infant health outcomes [14]. All interviews after delivery occurred within 2 years after birth; ~90% occurred within 6 months after delivery; another 9% occurred before the end of first year of life. The participants’ physicians were also interviewed after delivery when possible to obtain data on the occurrence of oral clefts and birth defects and abnormal delivery events. Participants and physicians were also interviewed after miscarriages/stillbirths to obtain data on birth defects. However, no data were available to measure the occurrence of birth defects and oral clefts in most miscarriage/stillbirth cases (there were only two stillbirth cases in the study). The study was designed from the beginning to focus on oral cleft occurrence in live births given the challenges of measuring these outcomes in miscarriages/stillbirths.

The OCPP aimed at observing 1,582 births (791 births in each folic acid group) in order to achieve 80% power for a 50% reduction in a baseline rate of 5% recurrence. The study assumed a birth rate of 0.0957 births per participant-year, which implied that about 16,531 participant-years were required to observe the 1,582 births.

### 2.2. Recruitment and Ethics Approvals

All potential participants were screened for eligibility using the same data forms and inclusion criteria across all sites by trained and qualified study staff before they were enrolled [14]. After confirming eligibility, the study staff asked the women to consent. For illiterate participants, confirmation was obtained as a thumbprint in the presence of a witness (only 10 participants reported no formal schooling). The study staff assured all women that refusal to participate in the study would in no way affect further treatment or care at the clinic. All interviews, informed consents, and data collection forms were in Portuguese. The study protocol, informed consent, manual of operations, and data collection forms were approved by the ethics committees of the study sites and by CONEP, the national committee of research ethics in Brazil.

### 2.3. Study Population

The study was conducted at six craniofacial clinics in Brazil (see detailed list in online Appendix). The clinics were selected based on their long experience in providing care to patients with oral clefts and most of them are considered referral centers for oral cleft care. Participants were identified from the population of patients served by these clinics. Enrollment was first initiated in 2004 using an outreach model for the patient pool served by the first study clinic. In that model, the study staff conducted meetings with the study participants in their local communities during which data were collected and study pills provided. In 2005, a clinic-based recruitment model was initiated at two new sites, where participants were recruited and interviewed at the study clinic. The clinic-based model collected data from participants over phone interviews and mostly delivered study pills via express mail; in-person follow-ups were completed with the participant every 6 months when possible. In 2007, the first site switched from the outreach model to a clinic-based model, and two new sites were added under the clinic-based model. A sixth site following the clinic-based model was added in 2008.

### 2.4. Inclusion Criteria

The study participants were women 16–45 years of age who have nonsyndromic/isolated oral clefts or had had at least one natural child of any age with nonsyndromic/isolated oral clefts who and who had received care at the study clinics [14]. The study participants also resided in the study catchment area consisting of the state where the clinic is located and surrounding states. During the first two years of the study (2004–2006) in the outreach model, both cleft lip with/without palate as well as cleft palate only were included in the study. Beginning in 2006, new recruitment was limited to cases of cleft lip with or without cleft palate, and cases with cleft palate only were no longer enrolled. The reason was to avoid any potential confounding effects of cleft palate only which is commonly separated from cleft lip with/without cleft palate in studies of cleft etiology, even though there may be overlap in etiology and recurrence prevention.

### 2.5. Exclusion Criteria

Participants who met any of the following criteria at the time of screening were excluded from participation [14]:

1. Syndromic/non-isolated cleft status including cases with recognized syndromes, cases with a chromosome abnormality, cases with one or more other major structural anomaly, cases with cognitive delay (IQ or equivalent less than 80), or cases exposed to phenytoin or valproic acid in utero.
2. Any first degree relative (that is a parent, sibling or child) who has cleft palate only (this was added in 2007 when new enrollment was limited to participants who are themselves affected or have affected children with cleft lip with/without palate).
3. The woman or her husband/partner was sterilized (such as tubal ligation).
4. Using intrauterine devices or injectible contraceptives (added in 2008).
5. Using anti-epileptic drugs (since the metabolism of anti-epileptic drugs requires a great deal of folic acid).
6. Using drugs that contain benzodiazepines (as these may increase the risk for birth defects and oral clefts).
7. Women who were pregnant at screening.
8. Women who were planning to move outside of the catchment area of the study within the next year.
9. Women who had B_12_ deficiency as determined from testing participants’ blood samples in the study before supplementation (B_12_ level below 174 pg/mL or 134.328 pmol/L), which may be masked by folic acid.
10. Women who were allergic to folic acid.

Participants who did not meet these criteria at enrollment because these were missed during the initial screening or the subject met these criteria after enrollment but were later found to meet the following exclusion criteria were discontinued from participation: definite sterilization of the woman and/or husband/partner, detecting B_12_ after randomization and a hematologist recommended that the woman be fully withdrawn from the study (no participants met this criterion), and taking epileptic drugs unless the participant is pregnant. B_12 _levels were assessed every 4 months between 2004 and 2006. After that, B_12 _levels were assessed once every 12 months.

### 2.6. Study Outcomes

The primary outcome of the study was the recurrence of isolated oral clefts, defined as the birth of a child with an oral cleft to the study women during their participation [14]. This is considered recurrence since only women who are themselves affected with oral clefts or have had a previous child with an oral cleft are enrolled in the study. The study infants were examined in person by a trained study staff to check and document oral clefting status and look for evidence of associated syndromes.

The study evaluated several secondary outcomes. The cleft recurrence rates in the OCPP were compared to historic recurrence rates. We calculated these rates by surveying women who are themselves affected or have had children affected with isolated cleft lip with/without cleft palate who were obtaining care from the same clinics involved in the OCPP but who did not enroll in the OCPP for reasons unrelated to cleft recurrence risk (such as sterilization and refusal to participate). Only cases with cleft lip with/without cleft palate were included in the historic recurrence survey; cases with cleft palate only were excluded. The study staff interviewed these women in 2011–2012 for their complete pregnancy history. We calculated the historic recurrence rates for the period after the initiation of the folic acid fortification program (after 2004) since the OCPP infants were born during this period and fortification may affect oral cleft recurrence. For affected women, the recurrence rate was defined as the proportion of affected children among all live births. For unaffected women with an affected child, the recurrence rate was defined as the proportion of affected children among all live births after the affected child.

The severity of oral clefts, birth weight, gestational age, and preeclampsia were also evaluated as secondary outcomes. In addition, we report here a comparison of head circumference, length at birth, and Apgar scores between the study births in the two folic acid groups.

### 2.7. Statistical Analysis

Outcomes were compared between the two doses of folic acid to which the women were assigned randomly at enrollment. Data for all women and infants were analyzed in the treatment arm to which the women were randomized, even when the women did not comply fully with the assigned treatment regimen as described above. We recognize that the sample size does not provide adequate power to identify moderate effects for small frequency outcomes when analyzing the live birth sample. Given the randomized design of the study, we compared the outcomes between the two folic acid groups using two-group comparison tests including a Fisher’s exact test for binary outcomes and a Student’s *t*-test for continuous outcomes. A Wilcoxon rank-sum (or Mann-Whitney) test was also used for the continuous outcomes since they did not meet normality tests (except for head circumference). Fisher exact tests and student’s t-tests were also used to compare the baseline characteristics between the two folic acid groups. We compared the OCPP recurrence rates to the historic rates using a one-sample *z*-test for proportions.

At the beginning of the study, there was no limit on how long a woman may participate. Participants were allowed to enroll again in the study in the same treatment group after their pregnancy if they wished to do so and if they still met all the eligibility criteria. Nine women participated a second time and had live births. In 2008, participation time was limited to three years. The study participants participated in the study until their delivery if they became pregnant or until they had completed three years of participation without becoming pregnant. After delivery, the participants were allowed to participate again if they had not completed a total of three years of participation in the study. We first report the analyses only for first-enrollments and first pregnancies. In additional analyses of cleft recurrence, we add women who participated a second time.

## 3. Results

### 3.1. Sample Description

**Figure 1:**
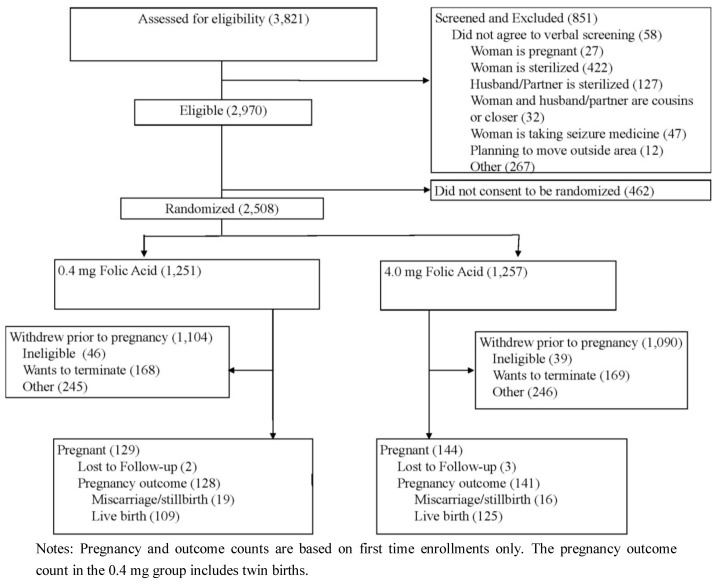
Study flow diagram.

Between January 2004 and May 2009, a total of 3,821 women were screened for eligibility into the study. Of these, 851 (22.3%) met one or more of the study exclusion criteria. Of the 2,970 eligible women, 2,508 women gave consent and were randomized into the study treatment and control groups; the 462 who were not randomized did not consent to participate in the study. Of the randomized women, 273 became pregnant during their first participation in the study. Of these pregnancies, there were 269 verifiable pregnancy outcomes (miscarriage, still birth or live birth); 234 were live births, 33 were miscarriages, and two were still births. Of these live births, 225 live births occurred in families where the previous affected child or affected mother had cleft lip with/without cleft palate (but not cleft palate only). Of the randomized women, 913 chose to discontinue their participation in the study before pregnancy, and five pregnant women were lost to follow up. On average, these women withdraw 20 months after randomization (ranging from 0 to 57). Figure 1 shows a flow diagram for study screening, enrollment/randomization, withdrawal, and pregnancy occurrence and outcome status. The average participation length was 20 months and ranged from 0 to 72 months.

In 2009, the DSMB overseeing the study with the funding institute (NIDCR) recommended that all new recruitment be halted and enrollment of non-pregnant women participating in the study be ended due to the lower than anticipated enrollment and pregnancy rates, which implied that the sample of infants needed to achieve 80% power could not be reached over the planned project period. Therefore, the enrollment of all non-pregnant participants was terminated at all sites after that. All pregnant women were followed according to the protocol and their babies examined in person by a trained study staff while maintaining the blinding of the subject and study staff to the folic acid group assignment.

Table 1 compares the distributions of baseline clinical, demographic and socioeconomic characteristics of all randomized women between the two folic acid groups. There were no statistically significant differences in these variables between the two folic acid groups. Of the randomized sample, about 41% were women who are themselves affected with oral clefts and had no children at the time of enrollment; about 59% were unaffected mothers with affected children; affected mothers with affected children were less than 1%. About 77% of the affected patients (either participants or their children) had cleft lip with palate, and about 21% had cleft lip only. The sample had overall low socioeconomic status with about 40% of the participants having only fundamental or no education and 51% having intermediate education; about 49% of the sample were employed. There were no significant differences between the two groups in baseline serum and RBC folate levels measured at enrollment before supplementation.

**Table 1:** Baseline confounders by treatment.

Demographic Variable		0.4 mg Folic Acid	4.0 mg Folic Acid	*p*-Value	Total
*Subjects, N*		*1,251*	*1,257*		*2,508*
*Cleft status group, n (%)*		*1,251*	*1,257*	*0.6437*	*2,508*
	Affected mother without children	502 (40.1)	518 (41.2)		1,020 (40.7)
	Unaffected mother of affected children	742 (59.3)	729 (58.0)		1,471 (58.7)
	Affected mother with affected children	7 (0.6)	10 (0.8)		17 (0.7)
*Family cleft type, n (%)*		*1,233*	*1,238*	*0.4733*	*2,471*
	Cleft lip	243 (19.7)	268 (21.6)		511 (20.7)
	Cleft palate	36 (2.9)	33 (2.7)		69 (2.8)
	Cleft lip with cleft palate	954 (77.4)	937 (75.7)		1,891 (76.5)
*Age, n (%)*		*1,243*	*1,253*	*0.5682*	*2,496*
	<20	229 (18.4)	204 (16.3)		433 (17.3)
	20–29	625 (50.3)	646 (51.6)		1,271 (50.9)
	30–39	356 (28.6)	370 (29.5)		726 (29.1)
	≤40	33 (2.7)	33 (2.6)		66 (2.6)
*Marital status, n (%)*		*1,247*	*1,257*	*0.6058*	*2,504*
	Single	422 (33.8)	414 (32.9)		836 (33.4)
	Married or stable relationship	801 (64.2)	812 (64.6)		1,613 (64.4)
	Divorced or widowed	24 (1.9)	31 (2.5)		55 (2.2)
*Highest level of schooling, n (%)*		*1,174*	*1,176*	*0.1423*	*2,350*
	Fundamental or none	432 (36.8)	427 (36.3)		859 (36.6)
	Intermediate	616 (52.5)	592 (50.3)		1,208 (51.4)
	University	126 (10.7)	157 (13.4)		283 (12.0)
*Employed in the past month, n (%)*		*1,174*	*1,176*	*0.4336*	*2,350*
	Yes	579 (49.3)	561 (47.7)		1,140 (48.5)
	No	595 (50.7)	615 (52.3)		1,210 (51.5)
*Smoke cigarettes, n (%)*		*1,247*	*1,257*	*0.8070*	*2,504*
	Yes	139 (11.1)	144 (11.5)		283 (11.3)
	No	1,108 (88.9)	1,113 (88.5)		2,221 (88.7)
*Drink alcoholic beverages, n (%)*		*1,246*	*1,257*	*0.1103*	*2,503*
	Yes	145 (11.6)	173 (13.8)		318 (12.7)
	No	1,101 (88.4)	1,084 (86.2)		2,185 (87.3)
*Multivitamin Use, n (%)*		*876*	*888*	*0.6271*	*1,764*
	Did not take multivitamin	801 (91.4)	825 (92.9)		1,626 (92.2)
	1 to 3 times a week	7 (0.8)	5 (0.6)		12 (0.7)
	4 to 6 times a week	2 (0.2)	3 (0.3)		5 (0.3)
	Every day of the week	66 (7.5)	55 (6.2)		121 (6.9)
*Baseline Serum Folate (ng/mL)*		*1,211*	*1,211*		*2,422*
	Mean (SD)	11.4 (5.1)	11.9 (8.8)	0.0650	11.6 (7.2)
	Median	10.7	10.7	0.8838	10.7
*Baseline Red Cell Folate (ng/mL)*		*1,150*	*1,156*		*2,306*
	Mean (SD)	606.7 (451.6)	595.9 (387.2)	0.5390	601.3 (420.5)
	Median	573.5	581.0	0.8658	577.5

Note: Some observations had missing data on certain measures.

### 3.2. Cleft Recurrence and Secondary Outcomes

Table 2 shows the cleft recurrence and type in the OCPP focusing first on families where the previously affected child or affected mother had cleft lip with/without cleft palate but not cleft palate only and on first-time enrollments. The cleft recurrence rate was 2.9% (three affected out of 105 births) in the 0.4 mg folic acid group and 2.5% (three affected out of 120 births) in the 4 mg group (*p* = 0.59 based on a one-sided Fisher’s exact test). The total recurrence rate combining the two folic acid groups was 2.7% (six affected out of 225 infants). One of the live births in the 4 mg group was diagnosed with Van-der-Woude syndrome (VWS), which is the most common syndromic form of oral clefting with a dominant genetic inheritance model [15] and about 50% recurrence risk.

**Table 2:** Cleft recurrence and types in OCPP.

Outcomes		0.4 mg Folic Acid	4.0 mg Folic Acid	Total
Infants delivered, n		105	120	225
Recurrence of oral clefts, n (%)		3 (2.9)	3 (2.5)	6 (2.7)
*Cleft type, n (%)*				
	Cleft lip only	0 (0.0)	1 (0.8)	1 (0.44)
	Cleft palate only	0 (0.0)	1 (0.8)	1 (0.4)
	Cleft lip with palate	3 (2.9)	1 (0.8)	4 (1.8)

Notes: This is based on first time enrollment births only where previous affected child or affected mother had cleft lip with/without cleft palate (but not cleft palate only). One case with cleft lip only in the 4 mg group had Van-der-Woude syndrome (missed at maternal screening); excluding this case results in recurrence of 1.6%.

VWS was not diagnosed when the mother of this case was screened for eligibility before enrollment and should have been excluded. Excluding the VWS case results in an isolated oral cleft recurrence rate of 1.6% (*p* = 0.44). All three cleft cases in the 0.4 mg group were cleft lip with palate compared to one case in the 4 mg group; the rates of cleft lip with palate rates were 2.9% *versus* 0.8% in the 0.4 and 4 mg groups, respectively. Adding the births from families where the previously affected child or affected mother had cleft palate only or including the babies of mothers who participated a second time (*i.e.*, only including cases that qualified because of the first affected child or mother having cleft lip with or without cleft palate) has virtually no effect on the estimates of oral cleft recurrence as only a few cases are added as shown in Supplementary online Table 1.1 (the same 6 affected cases were observed).

Table 3 shows the calculated recurrence rates in the historic control group. Data were obtained on 1,238 at-risk children born subsequent to an affected child (722 children) or to an affected mother (516 children).

**Table 3:** Historic recurrence rates in Brazil post-fortification.

	Group	Total births	Affected	Rate (%)
*Any period*				
	Sibling affected	722	48	6.65
	Mother affected	516	36	6.98
*Post-fortification period*				
	Sibling affected	278	18	6.47
	Mother affected	50	3	6.00

We compared the OCPP rates first to the recurrence rate in the total historic group, and then to the recurrence rate in children exposed to the fortification program during the first trimester of pregnancy in order to account for any change in recurrence with fortification since women were randomized into the OCPP after the beginning of the fortification. The overall recurrence rate was 6.65% for having a prior affected sibling and 6.98% for affected mothers; these rates were fairly similar when restricting the sample to children exposed to the fortification program (6.47% for a prior affected sibling and 6.0% for affected mothers). Based on these historic recurrence rates and the proportions of affected women *versus* unaffected mothers of affected children, we calculated the “expected” historic recurrence rates for the OCPP groups for the births from the first time enrollments and whose previously affected sibling or mother had cleft lip with/without cleft palate (but not cleft palate only) and compared them to the observed rates in Table 4. The expected recurrence rate among OCPP live births was about 6.8% when using the overall historic recurrence rate, and about 6.3% when using the post-fortification historic recurrence rate. These expected rates were more than twice as the observed ones for both folic acid groups and were significantly different from the observed rates in the OCPP folic acid groups both separate and combined (*p* = 0.0009 when comparing the post-fortification expected recurrence rate to the observed recurrence rate in the combined group). 

**Table 4:** Expected *versus* observed recurrence rates in OCPP.

RCT group		Expected historical recurrence rate (%)	Observed recurrence rate (%)	*p*-value
*Compared to overall historic recurrence rate*				
	0.4 folic acid group	6.8 ^a^	2.9	0.0172
	4 mg folic acid group	6.8 ^b^	2.5	0.0026
	Both 0.4 and 4 mg groups	6.8 ^c^	2.7	0.0001
*Compared to historic recurrence rate post-fortification*				
	0.4 folic acid group	6.3 ^e^	2.9	0.0379
	4 mg folic acid group	6.3 ^f^	2.5	0.0077
	Both 0.4 and 4 mg groups	6.3 ^g^	2.7	0.0009

Note: Expected historical rates are calculated based on the historical rates from the recurrence study weighted by the proportions of babies born into each group (affected and unaffected mothers). Observed rates are based on first time enrollment births only with previous affected child or affected mother with cleft lip with/without palate (but not cleft palate only).^ a ^((37 × 6.98) + (68 × 6.65))/105; ^b ^((54 × 6.98) + (66 × 6.65))/120; ^c^ ((91 × 6.98) + (134 × 6.65))/225; ^e ^((37 × 6.00) + (68 × 6.47))/105; ^f ^((54 × 6.00) + (66 × 6.47))/120; ^g ^((91 × 6.00) + (134 × 6.47))/225.

Table 5 compares the other secondary outcomes between the two folic acid groups. There are no significant differences in infant’s mean birth weight, gestational age, length at birth, head circumference, and apgar scores between the two folic acid groups. Virtually similar results for differences in birth weight, length at birth, head circumference, and apgar scores between the two folic acid groups were observed when adjusted for gestational age using regression analysis, which is expected since there is no significant difference in gestational age between the two groups (detailed regression results available from the authors upon request). There was no significant difference between the two folic acid groups in preeclampsia (4.8% *versus* 3.7% in the 4 and 0.4 mg groups, respectively).

**Table 5:** Secondary outcomes by treatment.

Outcomes		0.4 mg Folic Acid	4.0 mg Folic Acid	*p*-value	Total
*Birth weight (g), n*		*108*	*125*		*233*
	Mean (SD)	3,228.8 (443.7)	3,159.9 (508.2)	0.2753 (0.4287)	3,191.8 (479.6)
*Gestational age (weeks),*		*108*	*123*		*231*
	Mean (SD)	38.5 (1.6)	38.6 (2.1)	0.6590 (0.3929)	38.5 (1.9)
*Head Circumference (cm), n*		*87*	*102*		*189*
	Mean (SD)	34.1 (1.4)	34.1 (1.8)	0.9075 (0.8427)	34.1 (1.6)
*Length (cm), n*		*107*	*120*		*227*
	Mean (SD)	48.2 (2.5)	48.3 (2.6)	0.9036 (0.4521)	48.3 (2.6)
*Apgar score, n*		*73*	*86*		*159*
	Median	9	9	(0.0868)	9
	Interquartile range	9–10	9–10		9–10
	Min-Max	2–10	8–10		2–10
*Preeclampsia, n*		*108*	*125*	*0.755*	*233*
	Yes n (%)	4 (3.7)	6 (4.8)		10 (4.3)
	No n (%)	99 (96.3)	107 (95.2)		206 (95.7)

Notes: The outcomes are for first time enrollment births only. Some observations had missing data on certain outcomes. The *p* values from the Wilcoxon rank-sum test are in brackets.

### 3.3. Compliance

Based on the pill counts, median compliance was about 74% in both groups. The changes in blood folate levels also suggest good compliance with the study interventions. Based on a subgroup of participants with reviewed laboratory tests conducted at the laboratory at the study site at the Hospital de Clínicas de Porto Alegre for participants enrolled in the clinic-based model (total of 1,312 tests), mean post-supplementation serum folate levels were about 13.0 and 14.3 ng/mL in the 0.4 and 4 mg groups, respectively, representing an increase by about 1.7 and 3.1 ng/mL compared to the baseline levels (about 15% and 28%). The post-supplementation mean serum folate level was significantly higher in the 4 mg than 0.4 mg group (*p* < 0.0001). Similarly, post-supplementation mean RBC folate levels were about 716 and 793 ng/mL in the 0.4 and 4.0 mg groups (increase from baseline mean values of 712 ng/mL and 717 ng/mL, respectively, in this subgroup). Similar to serum folate, the post-supplementation mean RBC folate level was significantly higher in the 4 mg than 0.4 mg group (*p* = 0.0021).

## 4. Discussion

The study observes no difference in recurrence rates between the 4 mg and 0.4 mg groups. The small sample limits our ability to draw formal statistical inference on the effect of high dosage folic acid (4 mg) relative to low dosage (0.4 mg) on oral cleft recurrence based on comparing the recurrence rates between these two groups. With an observed recurrence rate of 2.9% in the 0.4 mg group, the current sample size provides 37% power to detect a 100% decrease—the maximum possible effect size —in recurrence in the 4 mg group based on a one-sided test (assuming that recurrence in the 4 mg group is equal to or less than that in the 0.4 mg) and a 5% type-1 error. However, at this sample size, the study has 80% power to detect a 6 percentage-point difference in recurrence between the two groups (*i.e.*, 8.5% recurrence in the 0.4 mg group relative to 2.5% recurrence in the 4 mg group) based on a two-sided chi-square test and 5% type-1 error. Therefore at the current sample size, the study does not have statistical power to detect a smaller difference (<6 percentage-points) in recurrence rates between the two folic acid groups; a larger sample is needed to achieve acceptable power. In contrast, there was a decline in recurrence in both OCPP groups separate and combined compared to historic control groups. The study has reasonable power (65%) to detect the observed difference in recurrence in the combined sample of 0.4 and 4 mg groups compared to the post-fortification historic recurrence rate reported in Table 4 using a two-sided test and a 5% type-1 error (power of 80% using a one-sided test). The recurrence rates that can be detected in the individual folic acid groups as different from the post-fortification historic recurrence rate (at 80% power and based on a one-sided test and 5% type-1 error) and can therefore be ruled out in this analysis are 1.6% in the 4 mg group and 1.4% in the 0.4 mg.

The decrease in recurrence in the 0.4 mg group compared to the historic rate (similar to the 4 mg group) suggests that this dose may be effective in reducing cleft recurrence risk. However, more research is needed to test this hypothesis and quantify the effect. The relatively similar recurrence rates between the two folic acid groups may be due to the potential ineffectiveness of the higher dose in further reducing recurrence relative to the lower dose. This may also be complicated by the participants’ use of prenatal vitamins and folic acid supplements on their own as well as their dietary folate consumption. About 40% of the participants (based on data for 207 live births) reported using multivitamins and/or folic acid supplements other than those supplied by the study during the month before pregnancy and/or during the first trimester (37% in the 0.4 mg and 43% in the 4.0 mg). Even though these additional folate sources would elevate the folate levels in both the 0.4 and 4 mg folic acid groups, they may result in an average folate intake in the lower dose group that is markedly higher than the 0.4 mg per day.

One limitation of the study is introducing some changes in recruitment strategies (such as limiting length of participation to 3 years) and inclusion/exclusion criteria (such as not including cleft palate only and excluding women using injectable contraceptives) while the study was ongoing. Another limitation is the potential over-estimation of population recurrence risk based on the historic control groups since these were identified through craniofacial clinics providing care to patients with oral clefts. Therefore, women who have experienced a recurrence may have been overrepresented. This highlights the need to calculate these rates using population-based registries; however, these resources are relatively underdeveloped in Brazil and are currently not available for such efforts. These limitations should be considered when evaluating the generalizability of the study results.

The study is the first to shed some light using a double-blinded randomized design on effects of high dosage folic acid on fetal development. The results suggest that high dosage folic acid does not compromise fetal growth or increase perinatal risks. These findings are consistent with previous observational study results suggesting positive effects of prenatal folic acid supplementation on development [16,17], and are opposite to recent results from a mice study reporting adverse effects on fetal growth. Furthermore, there were no significant differences in the rate and types of adverse events between the two groups (detailed results available upon request). Adverse events were regularly reported to the DSMB and IRBs and a member of the DSMB routinely reviewed all adverse events. There were no meaningfully elevated rates of adverse events in either group compared to expected population rates. These results suggest that high periconceptional folic acid supplementation may be a generally safe intervention for future studies.

## Appendix

### List of Study Clinics

- Hospital de Reabilitação de Anomalias Craniofaciais, Bauru, Sao Paulo
- Hospital Santo Antônio: Obras Sociais Irmã Dulce, Salvador, Bahia
- Instituto Materno Infantil Prof. Fernando Figueira, Recife, Pernambuco
- Centro de Atendimento Integral ao Fissurado Lábio Palatal, Curitiba, Paraná
- Hospital de Clínicas de Porto Alegre, Porto Alegre, Rio Grande do Sul
- Fundação para Reabilitação das Deformidades Crânio-faciais, Lajeado, Rio Grande do Sul

**Table 1.1:** Oral cleft recurrence outcomes for selected groups.

Outcomes		0.4 mg Folic Acid	4.0 mg Folic Acid	Total	
*Adding births from families where previously affected child or mother had cleft palate only*					
Recurrence of oral clefts, n (%)		109	125	234	
	Yes	3 (2.8)	3 (2.4) *	6 (2.6)	
	No	107 (97.2)	122 (97.6)	228 (97.3)	
*Including births from first and second time enrollments*					
Recurrence of oral clefts, n (%)		113	130	243	
	Yes	3 (2.7)	3 (2.3)	6 (2.5)	
	No	110 (97.3)	127 (97.7)	237 (97.5)	
